# The immune checkpoint molecules PD-1, PD-L1, TIM-3 and LAG-3 in diffuse large B-cell lymphoma

**DOI:** 10.18632/oncotarget.26771

**Published:** 2019-03-12

**Authors:** Benjamin J. Chen, Ravi Dashnamoorthy, Pallavi Galera, Vladislav Makarenko, Hong Chang, Srimoyee Ghosh, Andrew M. Evens

**Affiliations:** ^1^ Department of Pathology, University of Massachusetts Medical School, Worcester, MA, USA; ^2^ Division of Blood Disorders, Rutgers Cancer Institute of New Jersey, New Brunswick, NJ, USA; ^3^ Institute for Clinical Research and Health Policy Studies and the Biostatistics, Epidemiology, and Research Design (BERD) Center, Tufts Medical Center, Boston, MA, USA; ^4^ Tesaro, Waltham, MA, USA

**Keywords:** lymphoma, immunotherapy, immune checkpoint, TIM-3, LAG-3

## Abstract

Signaling through immune checkpoint receptors may lead to T-cell exhaustion and function as immune escape mechanisms in cancer. For diffuse large B-cell lymphoma (DLBCL), the mechanistic and prognostic importance of these markers on tumor cells and the tumor microenvironment remains unclear.

We determined the immunohistochemical expression of PD-1, PD-L1, TIM-3, and LAG-3 on tumor cells and on tumor infiltrating lymphocytes (TILs) among 123 DLBCL patients. TIM-3 showed positive staining on tumor cells in 39% of DLBCL cases and PD-L1 expression was noted in 15% of cases. Both PD-1 and LAG-3 were positive on tumor cells in a minority of DLBCL cases (8.3% and 7.5%, respectively), but were more widely expressed on TILs in a correlated manner. With median follow-up of 44 months (*n* = 70, range 5–85), 4-year progression-free survival (PFS) and overall survival (OS) rates were significantly inferior among DLBCL patients with high vs low/negative TIM-3 expression (PFS: 23% [95% CI 7% to 46%] vs 60% [95% CI 43% to 74%], respectively, *P* = 0.008; OS: 30% [95% CI 10% to 53%] vs 74% [95% CI 58% to 85%], respectively, *P* = 0.006). Differences in OS remained significant when controlling for International Prognostic Index in Cox regression analyses (HR 3.49 [95% CI 1.40–6.15], *P* = 0.007). In addition, we observed that co-culture of DLBCL cell lines with primed T cells in the presence of anti-LAG-3 and anti-TIM-3 induced potent dose-dependent increases in *in vitro* cell death via AcellaTox and IL-2 ELISA assays, suggesting potent anti-tumor activity of these compounds.

## INTRODUCTION

Significant advances have been made in the use of immuno-modulating therapies in the treatment of a range of human malignancies, most notably inhibitors of programmed cell death-1 (PD-1) in relapsed and refractory Hodgkin lymphoma (HL) [1, 2]. A number of hematologic malignancies, including HL and diffuse large-B cell lymphoma (DLBCL), express the PD-1 ligands, PD-L1 and PD-L2, presumably as a means of dysregulating the immune response through PD-1:PD-L1/L2 interactions between tumor cells and T lymphocytes leading to T-cell exhaustion [3–5]. Immunotherapeutic targeting of tumor cells *in vitro* with humanized antibodies to PD-1 or PD-L1 disrupt this interaction, thus restoring the anti-tumor activity of the T cells, forming the basis for this approach to immunotherapy [4]. Overexpression of PD-L1/L2 in lymphoma has been shown to occur through various mechanisms, including activation of JAK/STAT pathways, EBV-driven mechanisms, and 9p24.1 gene amplifications [6–8]. Expression of PD-L1 by DLBCL has been linked to inferior outcome, demonstrating the potential importance for both prognostic and treatment selection [9, 10]. Tumor infiltrating lymphocytes (TILs) in lymphoma have also been shown to frequently express the immune checkpoint molecule PD-1 [11, 12].

Two additional immune checkpoint molecules investigated in the context of cancer immunotherapy include TIM-3 (T cell immunoglobulin and mucin domain-containing protein-3) and LAG-3 (lymphocyte activation gene-3, CD223) [13, 14]. TIM-3 is a type I transmembrane protein expressed on several types of immune cells, most notably on CD4+ Th1 and CD8+ cytotoxic T cells, that functions to limit the duration and magnitude of T-cell responses [13, 15]. In the setting of human cancers, TIM-3 is expressed on the T cells found in a range of malignancies, including melanoma, lung cancer, hepatocellular, and colon cancer. In these tumors, TIM-3 expression is often associated with dysfunctional T-cell function, as well as poorer prognosis in some tumor types (reviewed in [13]). In hematologic malignancies, TIM-3 expression has been observed in adult T-cell leukemia/lymphoma and extranodal NK/T cell lymphoma [16, 17]. TIM-3 was also found to be increased in peripheral blood CD3+ T cells of patients with DLBCL, which was related to tumor stage and response to conventional chemotherapy [18, 19].

LAG-3 is a member of the immunoglobulin superfamily and functions as a negative regulator of T-cell homeostasis. Upregulated LAG-3 expression was originally discovered in activated CD4^+^, CD8^+^ and NK cell subsets [20]. LAG-3 binds to MHC class II at a higher affinity relative to CD4, while LAG-3 expressed in cytotoxic T and NK cells binds to LSECtin commonly expressed in various tumors, as well as normal hepatocytes [14]. LAG-3 has been shown to be expressed in TILs of several tumor types, including breast, ovarian, and lung cancers, often in connection with increased PD-1+ T cells [21–23]. In syngeneic mouse tumor models of fibrosarcoma or adenocarcinoma, a combination of anti-PD-1 and anti-LAG-3 antibodies had a synergistic effect on tumor growth inhibition. *LAG3*^*-/-*^*/PDCD1*^-/-^ double knockout mice have also shown enhanced clearance of and survival from multiple transplanted tumor types [24]. LAG-3 has been shown to be expressed by intratumoral T cells in cases of HL, particularly in areas rich in the malignant Reed-Sternberg cells [25]. In follicular lymphoma, a subset of intratumoral PD-1+ T cells were also found to be LAG-3+, which correlated with functionally exhausted T cells and inferior outcome [26].

Given the functions of TIM-3 and LAG-3 in controlling T-cell activation, these molecules are under consideration as alternate pathways for the regulation of tumor-induced immune evasion that could be blocked to promote T-cell mediated anti-tumor immunity. However, there remains a considerable lack of data related to LAG-3 or TIM-3 expression in DLBCL, as well as other tumors, despite a number of clinical trials for anti-LAG-3 or anti-TIM-3 therapies (as single agent or combinations) in solid tumors and hematologic malignancies. In the current analyses, we characterized the immunohistochemical expression of PD-1, PD-L1, LAG-3 and TIM-3 in tumor cells and TILs in a cohort of untreated DLBCL patients and correlated their expression with pathologic and clinical features. In addition, we evaluated the effects of investigational anti-PD-1, anti-LAG-3 and anti-TIM-3 antibodies in *in vitro* cytotoxic assays of tumor-primed T cells against DLBCL cell lines.

## RESULTS

### Expression of immune checkpoint receptors in DLBCL

**Figure 1:**
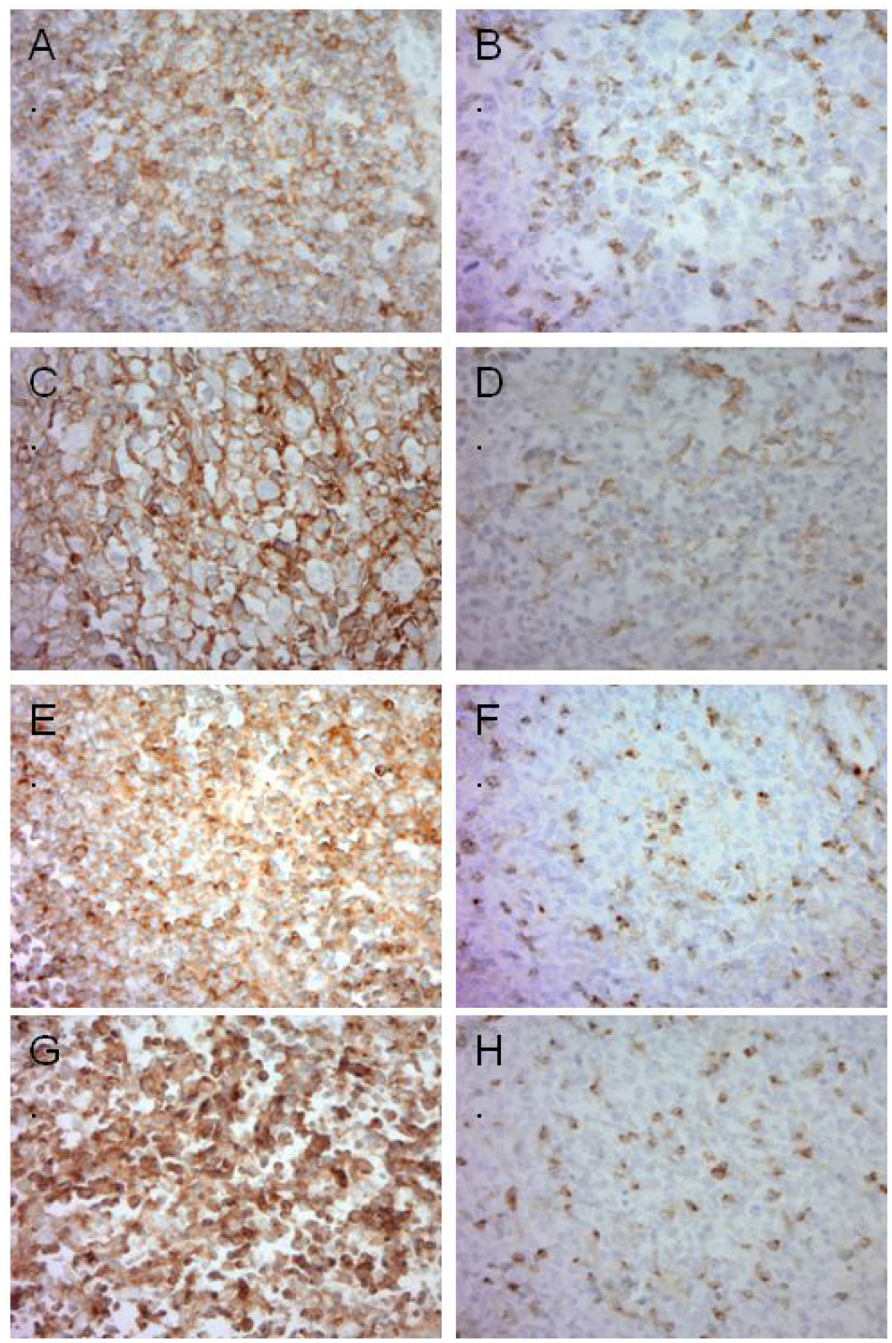
Representative images of immunohistochemistry in DLBCL (**A**) High PD-1 expression in lymphoma cells. (**B**) Negative PD-1 expression in tumor cells, positive in TILs. (**C**) High PD-L1 expression in lymphoma cells. (**D**) Negative PD-L1 expression in tumor cells, positive in TILs. (**E**) High TIM-3 expression in lymphoma cells. (**F**) Negative TIM-3 expression in tumor cells, positive in TILs. (**G**) High LAG-3 expression in lymphoma cells. (**H**) Negative LAG-3 expression in tumor cells, positive in TILs.

Tissue sections (whole sections and TMA) of newly diagnosed cases of DLBCL (*n* = 123) as described were examined for PD-1, PD-L1, TIM-3, and LAG-3 expression by immunohistochemistry. Representative photomicrographs of cases stained by IHC are shown in Figure 1. Staining results are summarized in Table 1. TIM-3 showed strong, membranous staining (TIM-3 score ≥80) on tumor cells in 39% of DLBCL cases (48/123). PD-L1 was expressed (≥30% tumor cells positive) in 15.6% of DLBCL (19/122), similar to previously published data from our group as well as others [9, 10]. There was a positive trend between TIM-3 and PD-L1 expression on tumor cells, but this was not statistically significant.

**Table 1:** IHC analysis of DLBCL cases

Marker		%
**PD-L1 (*n*** = **122)**	Tumor positive cases	15.6
	TIL/TAM positive cases	36.1
**TIM-3 (*n*** = **123)**	Tumor positive cases	39.0
	TIL positive cases	76.2
**PD-1 (*n*** = **120)**	Tumor positive cases	8.3
	TIL positive cases	77.0
	Avg positive TILs/HPF	31.8
**LAG-3 (*n*** = **120)**	Tumor positive cases	7.5
	TIL positive cases	84.7
	Avg positive TILs/HPF	46.1

Abbreviations: PD-L1, programmed cell death ligand-1; TIM-3, T cell immunoglobulin and mucin domain-containing protein-3; PD-1, programmed cell death-1; LAG-3, lymphocyte activation gene-3, CD223; TIL, tumor infiltrating lymphocytes; TAM, tumor associated macrophages; HPF, high power field; Avg, average.

Both PD-1 and LAG-3 were positive on tumor cells in only a minority of DLBCL samples (8.3% and 7.5%, respectively). Conversely, PD-1 and LAG-3 were widely expressed on TILs found in DLBCL in 77% and 84.7% of cases, respectively. For PD-1, the range of TILs per HPF was 0-250 with a median number of 15 and average of 31.8 positive lymphocytes/HPF. For LAG-3, the range of TILs per HPF was 0-200 with a median number of 40 and average of 46.1 positive lymphocytes/HPF. There was a positive correlation between counts for PD1+ TILs and counts for LAG3+ TILs (*r* = 0.32, *p* < 0.001). Counts for LAG3+ TILs also positively correlated with counts for TIM3+ TILs (*r* = 0.29, *p* < 0.001). TIM-3 status on tumor cells did not appear to correlate with PD1+ TIL or LAG3+ TIL counts.

### TIM-3 association with survival

**Figure 2:**
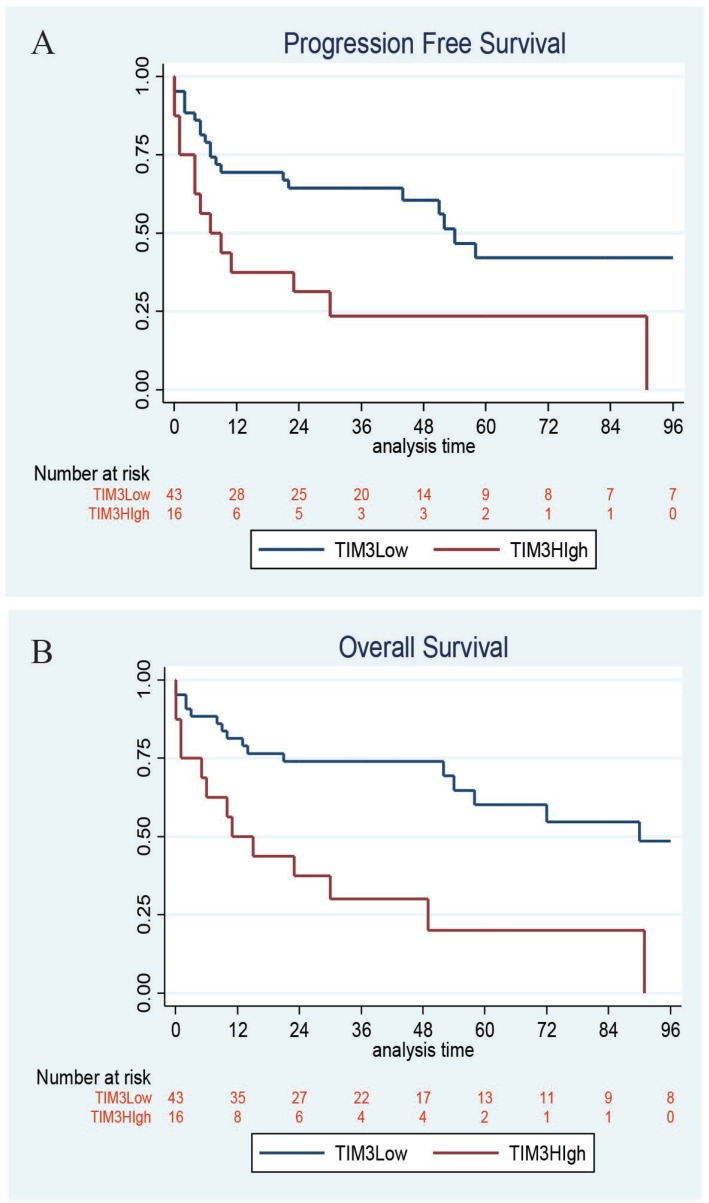
Kaplan–Meier survival High TIM-3 expression on DLBCL tumor cells correlates with inferior progression-free survival (PFS) with 4-year PFS 23% vs 60%, respectively, *P* = 0.008 and overall survival (OS) with 4-year OS 30% vs 74%, respectively, *P* = 0.006.

Clinicopathologic features, treatment, and outcome data were available for 70 patients (Table 2). The majority of patients received R-CHOP therapy (64%). With median follow-up of 44 months (range 5–85), the median PFS for all patients was 24 months, and the median OS for all patients was 29 months. Furthermore, the 4-year PFS and OS rates were significantly inferior among DLBCL patients with high (TIM-3 score ≥80) vs low/negative TIM-3 expression (PFS: 23% (95% CI 7% to 46%) vs 60% (95% CI 43% to 74%), *P* = 0.008; OS: 30% (95% CI 10% to 53%) vs 74% (95% CI 58% to 85%), *P* = 0.006) (Figure 2). When controlling for International Prognostic Index, the difference in PFS lost statistical significance (*P* = 0.10) while the difference in OS maintained statistical significance (HR 3.49, 95% CI 1.40–6.15, *P* = 0.007) in Cox regression analyses.

**Table 2:** Clinicopathologic characteristics of DLBCL patients

Characteristic		All patients	TIM3 low	TIM3 high	*P*
**Gender (*n*)**	Male	32	24	8	0.20^*^
	Female	38	23	15	
**Median Age (yrs)**		69	70	68	0.47^#^
**Performance Status (*n*)**	0–1	35	24	11	0.08^*^
	2+	17	11	6	
	NA	18			
**Stage (*n*)**	1-2	28	21	7	0.22^*^
	3-4	38	23	15	
	NA	4			
**IPI Score (*n*)**	0-2	33	26	7	0.02^*^
	3+	24	12	12	
	NA	13			
**Treatment (*n*)**	R-CHOP	45	31	14	0.68^*^
	Other	25	16	9	

^*^Chi-square test.

^#^*t*-test.

Abbreviations: TIM-3, T cell immunoglobulin and mucin domain-containing protein-3; n, number; yrs, years; GCB, germinal center; NA, not available; IPI, International Prognostic Index; R-CHOP, rituximab, cyclophosphamide, doxorubicin, oncovin, prednisone.

### Cell mediated cytotoxicity with anti-TIM-3 or anti-LAG-3

Considering that we observed PD-1, TIM-3 and LAG-3 expression in TILs, and TIM-3 expression in tumor cells of many cases of DLBCL, we next investigated the effects of blocking PD-1, TIM-3 and LAG-3 in the context of tumor-specific cytotoxic activity. For these experiments, we utilized isogenic CD8^+^ T cells and DCs to prepare tumor-specific cytotoxic T cells via DC-mediated priming as detailed in the Methods. Following 3 weeks of differentiation and maturation of DCs, and DC-mediated priming of T cells with irradiated lymphoma cell lines (Raji or SUDHL-10), the isolated T cells were investigated for cytotoxic activity against actively growing Raji or SUDHL-10 tumor cells, in the presence or absence of anti-PD-1, anti-TIM-3, or anti-LAG-3 experimental compounds. For these experiments, T cells were derived from 2 separate donors and used in similar sequential experiments.

**Figure 3:**
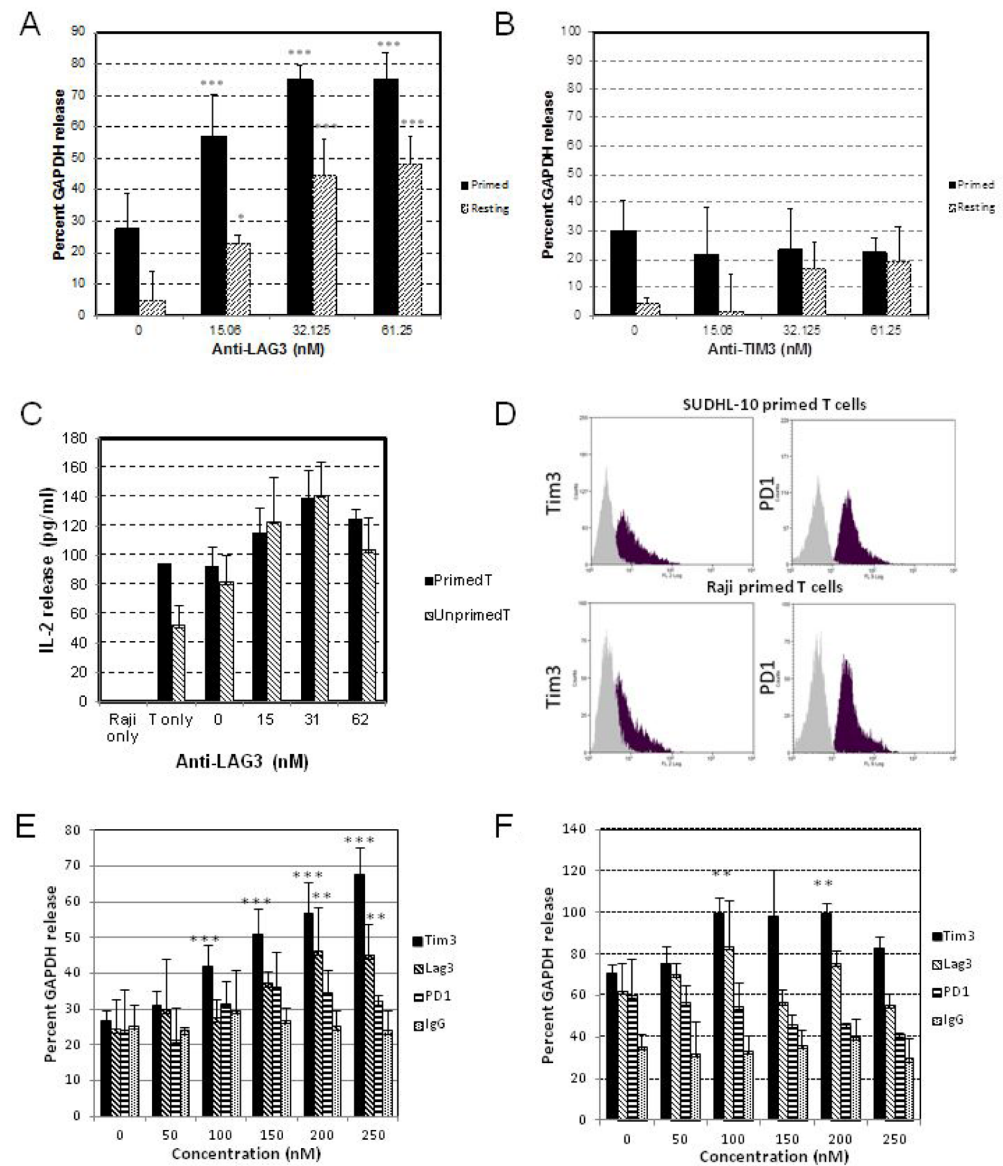
Co-culture of DLBCL and primed T cells Matured and differentiated isogenic dendritic cells (DC) were used to prime T cells derived from donor#1 using irradiated Raji cells for two weeks, with IL-2 supplementation. Primed T cells, unprimed T cells, at 4:1 ratio (T cells:Raji) were co-cultured with target cells for 4 hours ((**A**, **B**) for Acellatox GAPDH release assay) or 72 hours ((**C**) for IL-2 release assay). Matured and differentiated isogenic dendritic cells (DC) were used to prime T cells derived from donor#2 using irradiated SUDHL10 or Raji cells for two weeks, and isolated primed T cells were analyzed for PD-1 and TIM-3 expression by flow cytometry (**D**), or co-cultured with target cells at 4:1 ratio for 4 hours ((E) SUDHL10 or (F) Raji cells), and assayed for cell mediated cytotoxicity using Acellatox GAPDH release assay. (^**^denotes *P* < 0.05, ^***^denotes *P* < 0.001, compared with isotype IgG).

Results from our experiments with initial tumor-primed CD8^+^ T cells against Raji cells showed that anti-LAG-3, but not anti-TIM-3, induced a potent dose-dependent increase in cell-mediated cytotoxicity with 15 nM IC_50_ compared to 61.25 nM IC_50_ using unprimed/resting T cells, as determined by AcellaTox-Glo assay (Figure 3A, 3B). However, we observed a dose-dependent increase in IL-2 release from both primed and resting T cells with the addition of anti-LAG-3 in the presence of Raji cells, but not with anti-TIM-3 (data not shown), based on IL-2 ELISA assay (Figure 3C).

Using tumor-primed CD8^+^ T cells derived from a second donor and primed with either irradiated SUDHL10 or Raji cells, primed T cells showed increased TIM-3 and PD-1 expression compared to resting/unprimed CD8^+^ T cells, as determined by flow cytometry (Figure 3D), suggesting activation of immune checkpoints in these T cells. We then performed the AcellaTox cell-mediated cytotoxicity assay with anti-TIM-3, anti-LAG-3, anti-PD-1 and control IgG using these T cells. With anti-TIM-3, we observed a potent dose-dependent increase in the cytotoxicity of SUDHL10-primed compared to Raji-primed T cells against Raji cells (Figure 3E, 3F), despite both sets of tumor-primed T cells being TIM-3^+^ by flow cytometry (Figure 3D). With anti-LAG-3, a statistically significant difference in GAPDH release compared with control was observed at the highest concentration (>200nM) with SUDHL10-primed T cells, but not with Raji-primed T cells (Figure 3E, 3F). Additionally, there was no detectable presence of LAG3 by flow cytometry (data not shown), highlighting the variability between T cells primed with different antigens.

## DISCUSSION

DLBCL is an aggressive and heterogeneous non-Hodgkin lymphoma (NHL) that has been shown to respond in limited circumstances to anti-PD-1 monotherapy, while responses are more robust in HL. Checkpoint blockade for NHL, in general, has not shown consistent results, except in cases with genetically defined 9p24.1 amplification, such as primary mediastinal B-cell lymphoma [27], for which pembrolizumab was recently approved by the US FDA for treatment of patients with relapsed/refractory disease [28]. There remains much more limited data regarding the checkpoint molecules TIM-3 and LAG-3 in DLBCL. Overall, the heterogeneity of the expression of checkpoint molecules on DLBCL tumor cells and the microenvironment needs further study to determine mechanistic and prognostic features important for patient outcome, as well as to identify new immune targets for treatment. The goal of this study was to investigate the expression pattern of PD-1, PD-L1, TIM-3 and LAG-3 in DLBCL.

We demonstrate for the first time that TIM-3 is highly expressed in DLBCL tumor cells in over a third of cases (39%), as well as in TILs. We also found a correlation between high TIM-3 expression and worse overall survival in univariate analysis that persisted when controlling for IPI score. These data suggest that the tumor microenvironment in DLBCL is likely directly affected by TIM-3 expression by tumor cells and TILs leading to decreased immune surveillance and tumor clearance. This is analogous to the immunosuppressive concept of PD-L1 and PD-1 expression in tumors, particularly where PD-L1 expression in DLBCL was also correlated with worse overall survival [9, 10]. We found a trend in positive correlation between TIM-3 and PD-L1 expression on tumor cells that was not statistically significant in this cohort. Larger studies will be necessary to better understand the relationships between checkpoint molecule expression. The precise mechanisms of TIM-3 expression in DLBCL lymphoma cells, and the consequences of tumor versus TIL expression of TIM-3, are of interest to further understanding the importance of TIM-3 expression in lymphoma.

For PD-1 and LAG-3, we did not find a large number of cases with tumor cells expressing these markers; although a significant minority of cases with positive tumor expression showed strong expression. Interestingly, we found that almost all cases of DLBCL contained TILs that express PD-1 and LAG-3 in a correlated manner. Expression of these markers on TILs did not appear to correlate with patient outcome, nor did they appear to correlate with the expression patterns of PD-L1 or TIM-3 on tumor cells. The possibility, however, that expression of PD-1 and LAG-3 on TILs, as well as on tumor cells in a minority of cases, represents an indicator of immune dysregulation is certainly of interest. In future experiments, multicolor analysis and flow cytometry approaches will help further elucidate immune signatures that could further predict response (or resistance) to combination immunotherapy and prognosis. Co-expression of PD-1 and LAG-3 observed in exhausted TILs present in follicular lymphoma were found to be responsive to combined blockade of both PD-1 and LAG-3 [26]. Prior studies based on retrospective analysis reported a correlation between elevated expression of PD-1 and TIM-3 in CD3^+^ T cells with a poor response to standard chemotherapy in newly diagnosed DLBCL patients [18]. It was also reported that PD-1, TIM-3 and LAG-3 are elevated in a subset of BTLA^+^ T cells and correlated with poor prognosis in DLBCL [29]. These studies highlight the potential interplay among multiple checkpoint molecules in the pathogenesis of lymphoma.

In the *in vitro* studies, we demonstrated blocking activity by anti-TIM-3 and anti-LAG-3 compounds in our experimental model system consisting of tumor-primed cytotoxic CD8^+^ T cells. We found that the effect of the compounds was variable between the different batches of donor-derived T cells, lymphoma cells used for priming, and target lymphoma cell lines. While these data provide initial evidence that blockade of TIM-3 or LAG-3 may induce T cell anti-lymphoma activity, further comprehensive analysis of these effects are needed to fully define the biological activity and potential utility of these compounds to target DLBCL utilizing isogenic cytotoxic CD8^+^ T cells and tumor cells derived from lymphoma patients. Furthermore, patient-specific characteristics will be essential to identifying and developing optimal, personalized therapies.

## MATERIALS AND METHODS

### Patients

Patient cases of untreated DLBCL diagnosed between 2000 and 2014 were retrieved from the surgical pathology files and medical records. The study was approved by all Institutional Review Boards. All cases were diagnosed and classified according to 2008 World Health Organization criteria. One-hundred and twenty-three cases of patients with untreated DLBCL were identified, a subset of which were reported in a prior study [10]. Thirty-seven DLBCL cases were part of a tissue microarray (TMA), and 86 cases DLBCL were studied as whole sections. A subset of TMA cases were also studied as whole sections with similar results (data not shown). Original diagnostic pathology reports were examined for pathologic features including germinal center (GC) vs non-GC characterization.

Patient demographics, clinical data, treatment and outcome information were available among 70 untreated DLBCL cases. Clinical parameters included gender, age, stage, performance status, lactate dehydrogenase (LDH), bone marrow involvement, other extranodal sites involvement, International Prognostic Index (IPI), treatment history, the dates of disease progression, relapse or death and cause of death.

### Immunohistochemistry and evaluation

Immunohistochemistry using anti-PD-L1 (clone E1L3N, #13684, Cell Signaling Technologies (CST), Danvers, MA, USA), anti-PD-1 (clone EH33, #43248, CST), anti-TIM-3 (clone D5D5R, #45208, CST), and anti-LAG-3 (clone 17B4, #40466, Abcam, Cambridge, MA, USA) antibodies was performed on 5 μm-thick, formalin-fixed paraffin embedded (FFPE) tissue sections and TMA sections using a Dako Autostainer (Dako Corporation, Carpinteria, CA, USA) with antigen retrieval methods (0.01 M citrate buffer at pH 6.0) as described previously [10]. The UltraView Universal DAB Detection kit (#760-500, Ventana Medical Systems, Tuscon, AZ, USA) was used according to the manufacturer instructions. Counterstaining was done as part of the automated staining protocol using hematoxylin (#760-2021, Ventana Medical Systems).

All IHC-stained sections were evaluated and scored by two hematopathologists independently. Discrepancies in scoring (<10% of cases) were resolved by consensus conference between the two pathologists. For tumor cell staining (as identified by morphologic examination), intensity of PD-L1, PD-1, TIM-3, and LAG-3 was scored as follows: 0 (no staining), 1+ (weak), 2+ (moderate), or 3+ (strong). The percentage of tumor cells showing any positivity was recorded. For PD-L1, tumors were considered positive if ≥30% of tumor cells exhibited 2–3+ staining, as defined previously. [10] PD-L1+ non-malignant cells (TILs and tumor associated macrophages [TAM]) as a percentage of total tumor cellularity was also recorded [6, 10]. For PD-1 and LAG-3, ≥1+ intensity in ≥10% tumor cells was considered positive. Tumor infiltrating lymphocytes (TILs) that were positive for PD-1 or LAG-3 were enumerated by recording the average number of positive lymphocytes from 2-3 40x fields in an area of highest density. For TIM-3, multiplying the staining intensity by percent tumor cells staining positive produced a TIM-3 score ranging from 0-300. A TIM-3 score of =/>80 was considered positive. Positive controls including placenta for PD-L1 and tonsil sections for PD-1, TIM-3, and LAG-3 were included in each batch of IHC staining.

### Statistical analysis

Characteristics were compared using *t*-test for continuous variables for means, chi-square test for categorical variable distributions, or Wilcoxon rank-sum for medians. Covariates were collected and comprised the data set on which univariate analyses for PFS and OS were performed. PFS was calculated from the date of diagnosis to date of death or disease relapse/progression. OS was computed from the date of diagnosis to the date of death. Patients without PFS or OS events were censored at the time of last clinical follow-up. Survival analyses were performed regardless of amount or length of therapy received. Four-year PFS and OS rates were estimated through Kaplan-Meier method, while survival differences were assessed using the log-rank test. Multivariate associations between clinical and laboratory factors and survivals (PFS or OS) were derived using parametric survival modeling with Weibell distribution. Hazard ratios (HRs) and their 95% confidence intervals (CI) were reported. All statistical analyses were conducted with STATA v13 (StrataCorp LP, College Station, Texas). Statistical correlation between IHC results were determined using Pearson’s method with correlation coefficient *r* > 0.25 and *P* < 0.001 at 95% CI reported in the analysis conducted with Originlab (Northampton, MA, USA).

### Generation of tumor-specific cytotoxic T cells

Isogenic CD8^+^ T and monocytes, negatively isolated from two separate donors were purchased from Astarte Biologics, Bothell, WA, USA. To generate DCs, 0.5 × 10^6^ monocytes were cultured using ImmunoCult Dendritic Cell Culture Kit (Stemcell Technologies, Vancouver, Canada) in 24 well plates for 2 days, followed by medium supplementation with differentiation cocktail and incubation for 2 days, then a maturation cocktail supplied in the kit, following instructions provided by the manufacturer. Tumor priming was performed using irradiated lymphoma tumor cells (SUDHL10, derived from a germinal center DLBCL; or Raji, derived from a Burkitt lymphoma) prepared by exposing the tumor cells to 30 Gy γ-radiation using Cs-137 Shepherd irradiator (Tufts University) and culturing for an additional 6 hours. On day 7, 2.5 × 10^5^ DCs were co-cultured with irradiated tumor cells at a 1:1 ratio and maintained for 16 hours, followed by supplementation with maturation cocktail for an additional 24 hours. For T-cell stimulation, antigen-pulsed adherent DCs were rinsed with medium to remove irradiated tumor cell suspension and co-cultured with T cells at a 1:10 ratio in LGM-3 medium (Lonza, Walkersville, MD, USA) containing 5% human serum and IL-2 (10 U/ml). IL-2 was supplemented on days 12, 15 and 19, followed by harvesting T cells to perform cell mediated cytotoxicity assay on day 20.

### Cell-mediated cytotoxicity assay

T-cell mediated cytotoxicity was performed by using AcellaTox-Glo based GAPDH release assay, as per manufacturer provided instructions (Cell Technology Inc. Freemont, CA, USA). Briefly, 0.5X10^4^ SUDHL-10 or Raji cells were used as target cells with tumor-specific cytotoxic T cells as effector cells at a 1:5 ratio. T cells were pre-incubated with indicated concentrations of antibodies, cell culture supernatant was collected after 5 hours of co-culture and assayed as previously described [30].

### IL-2 ELISA

Briefly, 0.5 × 10^4^ SUDHL-10 or Raji cells were used as target cells with tumor specific cytotoxic T cells as effector cells at a 1:5 ratio. T cells were pre-incubated with indicated concentrations of antibodies, cell culture supernatant was collected after 24 hours of incubation and assayed for IL-2 release using Human IL-2 DuoSet ELISA (R&D Biosystems, Minneapolis, MN, USA) following manufacturer provided instructions. Anti-PD-1, anti-TIM-3, anti-LAG-3 compounds for *in vitro* assays were generously provided by Tesaro.

### Flow cytometry

PD-1 (D3W4U), TIM-3 (D5D5R), isotype rabbit IgG and anti-rabbit IgG (H+L) fragment Alexa Fluor 488 were purchased from Cell Signaling Technologies. 1 × 10^6^ cells were washed twice in ice-cold stain buffer (BD Bioscience, San Jose, CA, USA), centrifuged at 300 g at 4°C, and re-suspended in 100 mL of stain buffer and incubated for 20 minutes on ice with appropriate dilutions of antibody. Stained cells were washed twice and re-suspended in 500 mL of stain buffer and analyzed immediately using CyAn ADP flow cytometer with Summit software (Beckman Coulter, Pasadena, CA, USA).

## CONCLUSIONS

In conclusion, the IHC expression of the immune molecules PD-1, PD-L1, TIM-3, and LAG-3 were associated with heterogeneous profiles in DLBCL. High TIM-3 expression in tumor cells, found in over a third of DLBCL cases, was associated with inferior PFS and OS. Furthermore, by using tumor-primed cytotoxic T cells and anti-TIM-3 and anti-LAG-3 compounds in *in vitro* experiments, we found evidence of increased anti-tumor activity against DLBCL cell lines following checkpoint blockade. Altogether, these findings support the need for continued characterization of tumor immune profiles and delineation of the mechanistic basis for checkpoint inhibitors in DLBCL.

### Editorial note

This paper was accepted based in part on previous peer-review by another journal and the rebuttal and revision.

## Abbreviations

PD-1: programmed death receptor-1
PD-L1: programmed death receptor ligand-1
LAG-3: lymphocyte activation gene-3
TIM-3: T cell immunoglobulin and mucin domain-containing protein-3
DLBCL: diffuse large B-cell lymphoma
TIL: tumor-infiltrating lymphocyte
TAM: tumor-associated macrophage
PFS: progression free survival
OS: overall survival
HL: Hodgkin lymphoma
NHL: non-Hodgkin lymphoma
EBV: Epstein-Barr virus
TMA: tissue microarray
GC: germinal center
LDH: lactate dehydrogenase
IPI: international prognostic index
FFPE: formalin fixed paraffin embedded
IHC: immunohistochemistry
HR: hazard ratio
CI: confidence interval
DC: dendritic cell
ELISA: enzyme-linked immunosorbent assay
